# Factors Associated With the Recruitment of Primary Care Workforce in Rural Vermont: A Pilot Study

**DOI:** 10.7759/cureus.71941

**Published:** 2024-10-20

**Authors:** Kathleen Waeldner, Christopher P Kruglik, Julia Halvorson-Phelan, Joon Young Lee, Carolyn Taylor-Olson, Marty Hammond, Mark K Fung, Jan K Carney

**Affiliations:** 1 Division of Public Health, Robert Larner, MD College of Medicine, University of Vermont, Burlington, USA; 2 Internal Medicine, Brattleboro Memorial Hospital, Brattleboro, USA; 3 Health Committee, Windham Aging, Brattleboro, USA; 4 Department fo Clinical Pathology, Robert Larner, MD College of Medicine, University of Vermont, Burlington, USA

**Keywords:** access to health care, aging, primary health care, rural health, vermont

## Abstract

Objective

This study, conducted in collaboration with Windham Aging, aims to explore the key factors influencing the recruitment of primary care professionals (PCPs) in rural Vermont. It strives to contribute to the development of effective strategies to address the healthcare needs of rural communities, particularly focusing on the aging population.

Methods

The study employed a qualitative approach, involving structured interviews with Family Medicine residents and Doctor of Nurse Practitioner (DNP) students training at the University of Vermont Medical Center who were interested in pursuing primary care. The interview questions were developed after a roundtable discussion with rural Vermont PCPs. The data were analyzed using thematic, inductive methods.

Results

The study included 12 participants. Its findings challenge common assumptions about rural healthcare recruitment. While financial incentives are traditionally considered pivotal, this research revealed that personal and lifestyle factors play a more crucial role. Such factors include prior experiences in rural healthcare, preferences for rural living, and community attributes such as housing availability, educational facilities, and political landscape. These insights reveal a complex interplay of personal and community factors in the decision-making process.

Conclusions

To effectively recruit PCPs in rural areas of Vermont, strategies need to extend beyond financial incentives. Emphasizing rural exposure during medical education and fostering stronger ties with rural healthcare settings during clinical rotations are key. Understanding the varied motivations behind healthcare professionals’ choices is crucial for developing successful recruitment strategies in rural areas.

## Introduction

Rural regions grapple with a mounting challenge: the recruitment of primary care professionals (PCPs) [1-3]. Certain areas within the state of Vermont struggle to sustain an essential primary care workforce, including Windham County, home to the state's oldest population [4]. In response, Windham Aging was established to enable senior residents to age in place. However, a November 2022 report found that an insufficient number of PCPs impedes residents from aging in place [5].

The PCP workforce in Windham County is undergoing a demographic shift, with 48% of PCPs over 60 years of age, increasing the risk of significant gaps in essential healthcare services for rural residents [4]. Prior studies on PCP dynamics in rural U.S. locations have identified themes influencing recruitment strategies of PCPs in rural communities, including financial incentives, social dynamics, lifestyle preferences, and the role of community [6-8]. Our research team conducted a pilot qualitative study to investigate how personal experiences, lifestyle preferences, and community characteristics influence PCP recruitment decisions in rural Vermont, which may help develop educational strategies to enhance the recruitment of rural PCPs.

This research was previously presented as a meeting presentation at the 2023 Health Equity Summit on October 1, 2023, and as a poster at the 2023 American Medical Association Research Challenge Poster Symposium in Winter 2023.

## Materials and methods

From January to May 2023, in partnership with Windham Aging, our research team employed a qualitative methodology to understand the motivations behind where healthcare trainees, about to enter the workforce, wanted to work. Rural regions were classified based on the 2000 Office of Management and Budget Metropolitan Statistical Area Designation [9]. Project activities did not meet the federal regulatory definition of research requiring IRB review and approval under 45 CFR 46.201(d) according to the University of Vermont Research Protections Office IRB as this project was part of a "normal educational practices in an established educational setting" as a first-year medical student public health project. 

An interview with five practicing rural Vermont PCPs was conducted to understand the motivations and challenges of rural medical practice. Comprehensive interviews with Doctor of Nurse Practitioner (DNP) students and Family Medicine residents were then conducted at the University of Vermont Medical Center. All trainees interviewed were within two years of entering the workforce and expressed a commitment to a career in primary care. The team sought to identify trainees’ motivations toward or against a practice in rural Vermont. Structured, direct interviews were conducted by first-year College of Medicine students who recruited interviewees via outreach from their respective program directors, utilizing both in-person and Zoom formats. The Family Medicine residents were interviewed during an in-person teaching day in the medical school simulation lab. The DNP students were interviewed over Zoom as there was no centralized teaching day for the DNP students during the participant recruitment period. All participants within two years of entering the workforce were extended an invitation to participate in this study. The sample size was determined by participant availability. Interviews typically lasted 10 minutes and were recorded and transcribed using Zoom’s transcription service.

The subsequent interview protocol was developed based on existing literature on rural healthcare workforce recruitment and insights from the roundtable discussion. Questions were structured around four key themes: financial, social, lifestyle, and community. Participants were asked to rate various community factors on a Likert scale of 1 to 5: 1 indicated ‘not important’ and 5 indicated ‘very important’. Standard demographic data was collected from each participant. Additional open-ended questions captured the unique aspects of practicing in rural Vermont (Appendices). The Likert scale data were analyzed through calculated means in each category. 

The qualitative analysis of the interview transcripts was conducted independently by three research team members who initially performed open coding to identify key concepts. Subsequently, axial coding was employed to organize these codes into broader thematic categories within the predefined domains of financial, social, lifestyle, and community considerations. Axial coding was conducted collaboratively to ensure consistency and comprehensiveness in theme identification. A cross-verification step was included, wherein a subset of transcripts was independently analyzed by an external reviewer.

## Results

**Figure 1:**
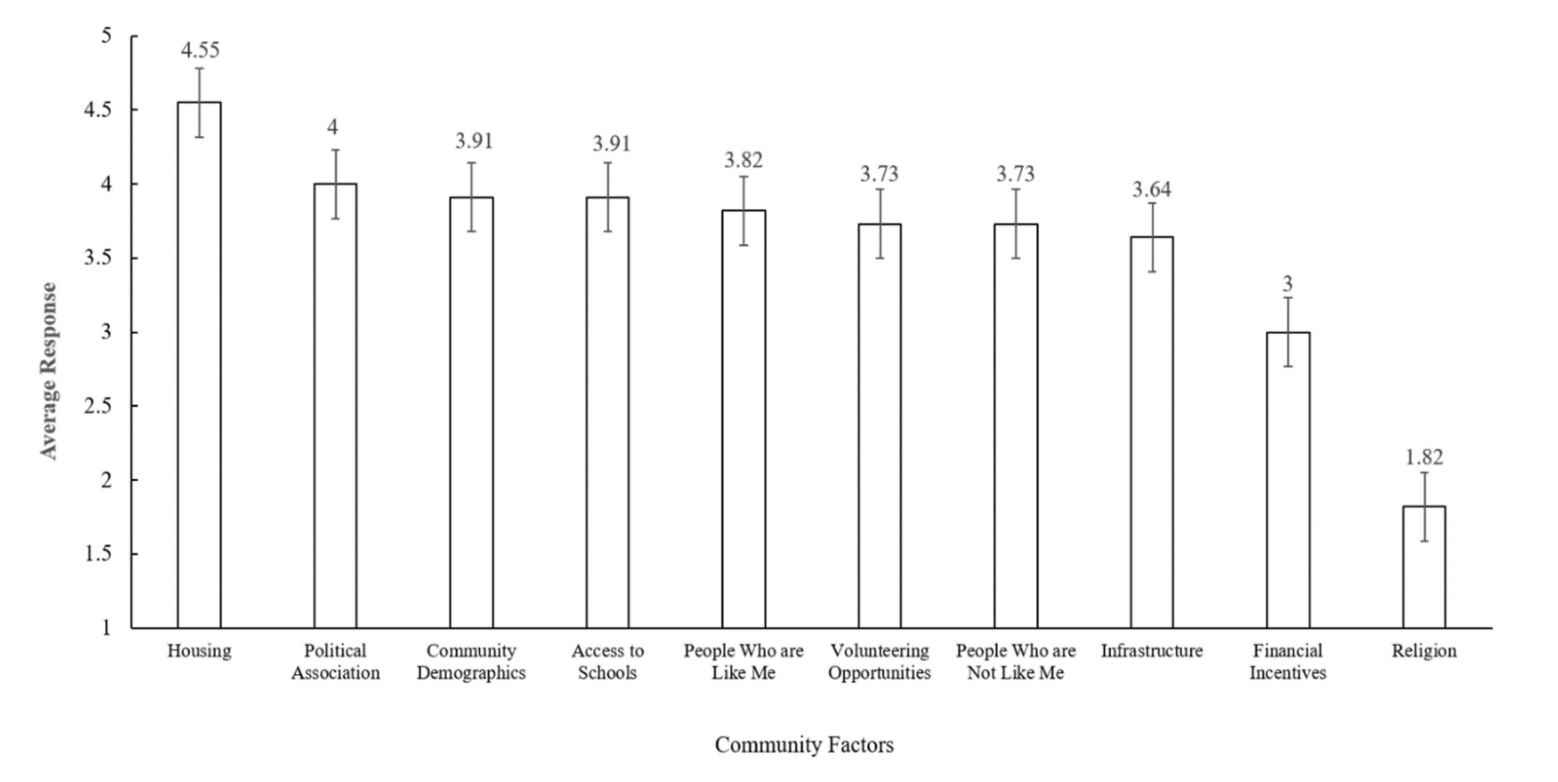
Ranked importance of community factors by study participants Participants ranked community factors on a scale of 1 to 5 with 1 representing "least important" and 5 representing "most important"

The response rate among Family Medicine residents was 75%, with eight out of 12 eligible participants responding. DNP students had a 9% response rate, with five out of 53 eligible participants responding (Table 1). The most important community attributes shared among participants included ‘Housing’ and ‘Political Association.’ The least important community attribute among participants was ‘Religion’ (Figure 1). Interviews revealed that financial incentives did not persuade trainees to work in rural settings. Exposure to rural health through clinical exposure was a common theme among those interested in pursuing rural health careers.

**Table 1:** Demographic characteristics and response rates among study participants

Variables	Values
Age, years, n (%)	
20-29	4 (30)
30-39	9 (70)
Average age, years	27
Gender assigned at birth, n (%)	
Male	1 (8)
Female	12 (92)
Race, n (%)	
White	9 (70)
Southeast Asian	2 (15)
Mixed race/ethnicity	2 (15)
Not reported	-
Profession, n (%)	
MD/DO resident	8 (62)
DNP student	5 (38)
Not reported	
Response rate, n (%)	
MD/DO resident	8 (75)
DNP student	5 (9)

## Discussion

Our results uncover the motivational landscape for new graduates inclined towards rural primary care in Vermont. The intrinsic rewards of community engagement and impactful clinical rotations significantly influenced trainees’ choices to practice in rural settings. A recurring theme among interviewees was the satisfaction of being part of a smaller community. Participants highlighted the unique rewards associated with rural practice, emphasizing the intangible benefits of community engagement over financial considerations. Similar findings had been noted in a previous study by Hustedde et al., which explored factors that encouraged female physicians to stay and practice in rural family medicine. The study showcased medical professionals’ need, search, and dependence on relationships built both in and out of their practice. Closely-knit rural communities nourished meaningful interhuman connections that out-incentivized financial gains for medical professionals [10].

While there were common threads in their responses, participants demonstrated a range of personal preferences driving their decision-making process regarding rural practice. Notably, housing affordability, community demographics, and access to schools ranked highly among participants’ considerations. The significance of the variety of personal factors affecting medical professionals’ decisions to move to new environments, as observed in this study, mirrored previous studies by Russell et al. and Davda et al. Specifically, these studies demonstrated that medical professionals moved for complex and individualized reasons that were ultimately rooted in personal values, upbringing, and vision of their careers and livelihoods [2,3].

MacKay et al. examined the complex factors influencing nurses’ decisions to practice rurally. Similar to our study, they found motivations to be complex and multifactorial. The authors proposed a “3P Model” theoretical framework to explore the interweaving of personal, professional, and place-related aspects of rural practice. The authors emphasize that each of these realms must be considered when formulating recruitment and retention initiatives [11]. Our study’s findings complement those of MacKay et al., and we believe training programs must seek to integrate each of the “3Ps” into their training and trainees’ exposure to rural care [11].

Abelsen et al. developed a similar framework to generate a stable rural workforce. This framework included “Five Conditions for Success” for recruitment and retention of primary care professionals, which include components such as active community participation, recognition of unique rural issues, and continuous monitoring and evaluation [11,12]. This framework moves beyond Vermont’s current policy initiatives of financial incentives for future providers and encourages an equitable approach to rural care by involving communities and continuously monitoring for success. Findings from our study demonstrate that trainees value the multifaceted approach employed by Abelsen et al. as they are motivated by a range of rural traits, rather than financial gain.

Participants’ experiences with rural clinical rotations during training proved formative in their decisions to practice in rural settings. One participant noted, "[Having a clinical rotation in rural Vermont] at least introduced me to a different way of practicing medicine... [Practicing in a rural setting] makes you think differently and a little bit more creatively." This sentiment suggests that exposure to rural healthcare environments during training can catalyze rural practice post-graduation. Holst’s integrative review found a positive correlation between rural placements during undergraduate medical training and later rural practice, affirming the sentiments of participants in our study [13].

Ogden et al. went a step further to demonstrate that the increased length of rural training during undergraduate medical education had a stronger correlation with later rural practice [5]. The study found a significant increase in entrance to rural practice by practitioners who completed lengthy rural rotations during training. Medical and DNP schools and training programs should work to place trainees in the same rural health placements during each rural health block, thereby allowing trainees to develop closer relationships with current rural professionals and with rural communities.

Throughout our study, participants repeatedly de-emphasized financial incentives as a primary driver for a rurally-focused career. Thus, financial compensation is just a piece of the puzzle. Despite states’ assumptions that financial considerations would play a pivotal role, our findings indicate that the allure of rural medicine is not primarily rooted in financial incentives but, rather, complex personal factors [14].

Finally, the literature suggests that trainees’ personal qualities influence their preference toward family medicine and their decision to practice rurally. Gill et al. found four factors that were significantly associated with trainees’ deciding on family medicine, rather than another specialty: emphasis on continuity of care (87.3 vs. 45.3%, p<0.001); length of residency (73.4% vs. 25.9%, p<0.001); the influence of family, friends, or community (67.1% vs. 50.2%, p=0.011); and preference for working in a rural community (41.8% vs. 10.9%, p<0.001). The study also found that trainees who decided to practice rurally were less motivated financially than their counterparts who decided to practice family medicine in urban settings [15].

One of this study's main limitations is the number of participants included in the interviews. The underrepresentation of the DNP student group skews the data to rely heavily on Family Medicine resident responses, which may introduce biases and limit the depth of insights into the motivations and considerations of PCPs entering the workforce. For example, DNP students may be motivated by slightly different reasons than Family Medicine residents, and these differing motivations could be useful knowledge in policy interventions to increase PCP recruitment in rural areas. Furthermore, the study's reliance on the University of Vermont Medical Center’s Family Medicine residency program as the primary source of participants might introduce a programmatic bias in ways that might not be reflective of PCPs from other training programs or institutions. Furthermore, Vermont is a predominantly rural state, and trainees may rank a Vermont training program highly for this reason. 

Hence, future studies should aim to diversify study participants in terms of both training program type and geographic location. Expanding respondent type in these ways will limit the reliance on Family Medicine residents and increase generalizability beyond the state of Vermont, increasing external validity. Future steps should prioritize connections between academic institutions and rural clinics. Collaborative efforts could include further development of structured programs, partnerships, or internships, fostering increased engagement between healthcare trainees and rural practitioners. Increasing these opportunities would not only expose more students to the realities of rural medicine but also provide rural clinics with the opportunity to actively participate in shaping the training experiences of the next generation of PCPs. Studies show that medical schools in rural areas are more likely to graduate physicians who will work in rural areas [13].

Implementing a comprehensive approach to integrating rural settings into clinical rotations, beyond the current practice, could be instrumental. This may involve the development of dedicated rural health tracks, immersive experiences, or extended rotations. Further, Family Medicine residency programs and DNP programs could expand their rural clinic site placements, ensuring rural healthcare exposure for all trainees by adding rural care as a required part of the curriculum. Exploring alternative channels, such as collaborations with professional organizations, can help overcome barriers to participation. Future studies could investigate whether coming from a rural versus urban background influences eventual practice location. Budhathoki et al. reached a more definitive conclusion that coming from a rural background was a powerful motivator for future practice. Differences in responses were noted in terms of a student growing up in a rural versus urban area [16].

## Conclusions

Our study reveals that community attributes and training exposure through clinical rotations play pivotal roles in shaping the preferences of emerging healthcare professionals in Vermont. Contrary to conventional assumptions, financial incentives did not emerge as the primary driver for new graduates seeking to practice primary care in rural Vermont. Instead, the allure of rural medicine is intricately intertwined with the sense of belonging in a smaller rural community and with exposure to rural care during training. Our study can inform targeted interventions to bolster the recruitment of rural professionals in Vermont. By enhancing connections between academic institutions and rural clinics, we can facilitate meaningful exposure to rural medicine, a strategic investment in healthcare professionals and rural communities who strive to age in place.

## Appendices

Interview guide

You are being asked to participate in this research project because you have been identified as a healthcare trainee who is only two years or less out of working to your full capacity in the field of primary care. The interview should take about 20-30 minutes and with your permission, it will be digitally recorded using Zoom’s transcription services to ensure all key points are accurately documented. Any identifying information you use in our discussion will be removed from the interview transcripts. If you wish to end the interview before I have asked all the questions or withdraw from the study, you are free to do so. 

It is important to note that there are no right or wrong answers to the questions and that no one will know what your specific answers were. Do you give your consent to move forward with the interview? Do you have any questions before we begin? 

Note to the interviewer: depending on how the participant responds to questions, the order in which these questions are asked can vary. 

Intake

1. What is your gender? 

2. How do you describe your sexual orientation or sexual identity? 

3. To which racial or ethnic group(s) do you most identify? 

4. What is your current age in years? 

5. What is your profession? 

6. If clarification is needed, ask the participant if they are in the Family Medicine residency program or Doctor of Nurse Practitioner program. 

7. Do you want to work in primary care once you finish your training? 

Community Factor Rankings

Please rate the following factors in terms of importance regarding the type of community where you want to live and work. Rate each factor on a scale of 1 through 5; 1 indicates the factor is not important at all, 2 indicates somewhat important, 3 indicates the factor is important, 4 indicates the factor is very important, and 5 indicates the factor is extremely important.

1. Volunteer and/or community opportunities

2. Presence of religious communities

3. People who are not like me 

4. People who are like me 

5. Financial incentives to work in the community 

6. Community demographics 

7. Infrastructure 

8. Housing availability 

9. Access to schools

10. Politics/policy 

Open-Ended Questions

Financial:** **How do financial incentives and compensation influence your decision to practice rurally? Would this persuade you to work rurally? 

Lifestyle:** **Do other people in your life influence your decision to practice medicine in a rural area?

Social:** **When thinking of a community in which you would like to live and work, how would you rank each of the following? 

Uncategorized/Other: Does your clinical training/residency experience influence your decision to work in a rural community? Does this experience influence the kind of medicine you want to practice? 

Did you grow up in a rural area, suburban area, or urban area? If you are interested in practicing in a setting different from where you grew up, can you explain why? If you are interested in practicing in a setting that is similar to where you grew up, can you explain why? 

Conclusion

Thank you for your time today. If you want a copy of the transcript, you can request one now or later.

## References

[1] Russell D, Mathew S, Fitts M (2021). Interventions for health workforce retention in rural and remote areas: a systematic review. Hum Resour Health.

[2] Davda LS, Gallagher JE, Radford DR (2018). Migration motives and integration of international human resources of health in the United Kingdom: systematic review and meta-synthesis of qualitative studies using framework analysis. Hum Resour Health.

[3] Wasko K, Jenkins J, Meili R (2014). Medical practice in rural Saskatchewan: factors in physician recruitment and retention. Can J Rural Med.

[4] Cameron PJ, Este DC, Worthington CA (2012). Professional, personal and community: 3 domains of physician retention in rural communities. Can J Rural Med.

[5] Ogden J, Preston S, Partanen RL, Ostini R, Coxeter P (2020). Recruiting and retaining general practitioners in rural practice: systematic review and meta-analysis of rural pipeline effects. Med J Aust.

[6] Koebisch SH, Rix J, Holmes MM (2020). Recruitment and retention of healthcare professionals in rural Canada: a systematic review. Can J Rural Med.

[7] Stenger J, Cashman SB, Savageau JA (2008). The primary care physician workforce in Massachusetts: implications for the workforce in rural, small town America. J Rural Health.

[8] Danish A, Blais R, Champagne F (2019). Strategic analysis of interventions to reduce physician shortages in rural regions. Rural Remote Health.

[9] (2024). United States Census Bureau: Metropolitan and Micropolitan. https://www.census.gov/programs-surveys/metro-micro.html.

[10] Hustedde C, Paladine H, Wendling A, Prasad R, Sola O, Bjorkman S, Phillips J (2018). Women in rural family medicine: a qualitative exploration of practice attributes that promote physician satisfaction. Rural Remote Health.

[11] MacKay SC, Smith A, Kyle RG, Beattie M (2021). What influences nurses' decisions to work in rural and remote settings? A systematic review and meta-synthesis of qualitative research. Rural Remote Health.

[12] Abelsen B, Strasser R, Heaney D (2020). Plan, recruit, retain: a framework for local healthcare organizations to achieve a stable remote rural workforce. Hum Resour Health.

[13] Holst J (2020). Increasing rural recruitment and retention through rural exposure during undergraduate training: an integrative review. Int J Environ Res Public Health.

[14] (2024). Vermont Department of Health: loan repayment and scholarships. https://www.healthvermont.gov/systems/health-professionals/loan-repayment-scholarships.

[15] Gill H, McLeod S, Duerksen K, Szafran O (2012). Factors influencing medical students’ choice of family medicine: effects of rural versus urban background. Can Fam Physician.

[16] Budhathoki SS, Zwanikken PA, Pokharel PK, Scherpbier AJ (2017). Factors influencing medical students' motivation to practise in rural areas in low-income and middle-income countries: a systematic review. BMJ Open.

